# Training and external validation of machine learning supervised prognostic models of upper tract urothelial cancer (UTUC) after nephroureterectomy

**DOI:** 10.1038/s41598-025-29043-w

**Published:** 2026-01-22

**Authors:** Rossella Nicoletti, Nick Ho, Hsiang-Ying Lee, Wen-Jeng Wu, Ekaterina Laukhtina, Pietro Spatafora, Chris Ho-Ming Wong, Ivan Ching-Ho Ko, Chi-Ho Leung, Gianluca Giannarini, Nikhil Vasdev, Paolo Gontero, Chi-Fai Ng, Ching-Chia Li, Wei-Ming Li, Hung-Lung Ke, Hsin‑Chih Yeh, Riccardo Campi, Sergio Serni, Mauro Gacci, Shahrokh Shariat, Thomas Choi, Jeremy Yuen-Chun Teoh

**Affiliations:** 1 S.H. Ho Urology Centre, Department of Surgery, Faculty of Medicine, The Chinese University of Hong Kong, Sha Tin, Hong Kong; 2 Department of Experimental and Clinical Biomedical Science, University of Florence, Florence, Italy; 3 The Hong Kong Polytechnic University, Hung Hom, Hong Kong; 4 Kaohsiung Medical University Hospital, Kaohsiung, Taiwan; 5 Department of Urology, School of Medicine, College of Medicine, Kaohsiung Medical University, Kaohsiung, Taiwan; 6 Department of Urology, Medical University of Vienna, Vienna, Austria; 7 Unit of Urology, Santa Maria della Misericordia Academic Medical Center, Udine, Italy; 8 Department of Urology, Hertfordshire and Bedfordshire Urological Cancer Centre, Lister Hospital, Stevenage, UK; 9 School of Life and Medical Sciences, University of Hertfordshire, Hatfield, UK; 10 AOU Città della Salute e della Scienza di Torino, Torino School of Medicine, Torino, Italy; 11 Department of Urology, Kaohsiung Medical University Gangshan Hospital, Kaohsiung, Taiwan; 12 Department of Urology, Weill Cornell Medical College, New York, USA; 13 Department of Urology, University of Texas Southwestern, Dallas, USA; 14 Hourani Center of Applied Scientific Research, Al-Ahliyya Amman University, Salt, Jordan

**Keywords:** Upper tract urothelial cancer, Nephroureterectomy, Machine learning, Cancer, Surgical oncology, Machine learning, Oncology

## Abstract

The European association of Urology (EAU) suggests a prognostic stratification of Upper Tract Urothelial Cancer (UTUC) based on high and low risk patients, with Radical nephroureterectomy (RNU) and bladder cuff resection being the gold standard for the treatment of non-metastatic High risk UTUC. However, no consensus on post-operative patient management or tools that predict who would benefit the most from a close follow-up rather than adjuvant chemotherapy regimen exist. in Machine Learning (ML) is gaining interest in Urology providing models for prognostic prediction purpose; It’s role in UTUC has not yet been investigated. We aim to develop and validate multiple supervised ML models based on patient- and tumor- related features to predict prognosis in patients with preoperative Histological or Imaging proved UTUC treated with RNU within a multiethnic large cohort. Data from an international multicenter large cohort of histologically proven UTUC patients from Asia and Europe treated with RNU were retrospectively collected. Twenty different ML-supervised predictive models were first trained and then external validate with two separate set. Nomograms were constructed based on 8 independent prognostic factors (age, gender, grading, pT, pN, presence of Carcinoma in Situ (CIS), multifocality and Lymphovascular invasion(LVI)) to predict 6 Outcomes (Overall Survival (OS), Cancer Specific Survival (CSS) and Disease Free Survival (DFS) at 3 and 5 year). Performances were compared using Area-under-curve (AUC) of Receiver-Operating Characteristics (ROC). A total of 3129 patients were enrolled: 637 Asian Patients (training cohort) and 2492 European patients (validation cohort). Upon training assessment, LR models achieved the best results, being the best model for prediction of 4/6 outcomes, with the best result in CSS both at 3 and 5 years (AUC: 0.85, 0.84, 0.81 for CSS-3y, CSS-5y and DFS-3y respectively). Upon external validation, LR(CSL) models achieve the best results, being the number 1 model for prediction of 3/6 outcomes (AUC: 0.84, 0.79, 0.77 for CSS-3y, OS-3y and OS-5y respectively). ML is a promising technology in the field of UTUC. Our model achieve favorable results in terms of prediction of prognosis after RNU, especially in terms of CSS at 3 and 5 years, moreover is the first model of prognosis taking into account the differences in epidemiology existing between European and Asian patients. Further clinical validation and verification of its reliability for the case selection of adjuvant therapy are needed to assess its use in clinical practice linked to clinical decision making. ML is an advancing technology in the field of medicine and urology, which can also be applied to the definition of the prognosis of patients with UTUC undergoing RNU. Our study represents the first experience investigating this potential.

**Supplementary Information:**

The online version contains supplementary material available at 10.1038/s41598-025-29043-w.

## Introduction

Urothelial carcinoma of the upper urinary tract (UTUC) is a rare disease, accounting for about 5–10% of all urothelial carcinomas^1^.The European association of Urology (EAU) suggests a prognostic stratification of UTUC based on high and low risk patients, with Radical nephroureterectomy (RNU) and bladder cuff resection being the gold standard for the treatment of non-metastatic High risk UTUC^2^. However, no consensus on post-operative patient’s management or risk stratification exist. The POUT trial, a phase III prospective randomized trial, aiming to evaluate the benefit of adjuvant chemo after RNU vs. surveillance in patients with pT2–T4 pN0–N3 M0 or pTany N1–3 M0 disease, in his preliminary subgroup analysis, demonstrated large variability in the benefits of patients undergoing adjuvant chemotherapy, underlining the need for a stratification strategy after RNU, especially in advanced disease setting^3^.

Several Prognostic nomograms, based on pre-operative and post-operative factors have been described^4–10^. However, to date none of them is currently used nor recommended by guidelines, with only the Yates et Al’s model (a nomogram to predict CSS post RNU) externally validated, using 200 bootstrap resamples^11^. Moreover, most of this nomograms didn’t take into account the existing differences in Patients with Asian ethnicity, who seem to present with more advanced and higher-grade diseases compared to other ethnicities^12^. This could led to models that are not Globally generalizable, as trained and tested only in a single-ethnicity population.

Over the past years, Machine learning (ML)-assisted models have been proposed as a supplement or alternative for standard statistical techniques, opening up the possibility of creating non-linear predictive models and with the ability to improve automatically^13^. In ML, through the process of supervised machine learning, it is possible to build a model using computer algorithms by making them learn the relationship between input variables (characteristics) and outputs (labels). In a first phase, the algorithms analyze the relationship between input and output thanks to a training dataset. Then, the algorithms are applied to a second set of data known as a validation set, to assess how well the predictive model is able to test inputs to predict outputs^14^.

Various ML techniques have been used in the field of Urology and especially in urothelial carcinomas. However, most of them were used in the lower urinary tract setting while the possible application of artificial intelligence within the field of the UTUC still remain unexplored, especially as a tool for prognosis prediction.

We aim to develop and compare training and validation performances of multiple supervised ML models based on patient- and tumor- related features to predict oncological outcomes [overall survival (OS), Cancer-specific Survival (CSS) and Disease-free survival (DFS)] in patients with preoperative Histological or Imaging proved UTUC treated with RNU within a large cohort of multi-ethnic patients.

## Matherial and methods

### Data sources

Data from an international multicenter large cohort of patients with preoperative Histological or Imaging proved UTUC treated with RNU between December 2001 and August 2020 were retrospectively collected in two dedicated database: the training cohort, consisting of Asian patients and the validation cohort, entailing of European Patients. Baselines as well as tumor related characteristics of patients were collected.

The common inclusion criteria were: patients undergoing RNU, with preoperative Histological or imaging proven UTUC, for which patient- and tumor-related data and oncological outcomes data were available.

### Features and outcomes of interest

We used a total of 8 features as input sourced among the listed patient and tumor related factors on EAU UTUC guidelines: age, gender, grading (according to World Health Organization (WHO) 1973 classification for patienst enrolled before 2004 and to the WHO 2004 classification for patients enrolled after 2004), pT, pN, presence/absence of Carcinoma In Situ (CIS), multifocality and presence/absence of Lymphovascular invasion.

The outcomes of interest were: overall survival (OS), cancer-specific survival (CSS) and disease-free survival (DFS) as defined as both local or intravescical recurrence both at 3 and 5 years from index, defined as date of RNU. Patients were followed up as appropriate, following the principles of EAU-Guidelines in all the Centers involved in the analysis.

### ML-supervised models

In this study, 20 predictive models were built using supervised learning algorithms, including logistic regression (LR), decision tree (DT) and its ensemble learning variants, support vector machines (SVM), k-nearest neighbours (KNN), and hard-voting ensemble of these algorithms. LR performs binary classification by modelling the relationship between input features and outcome with sigmoid function^15^. DT predicts an outcome by traversing a tree-like flowchart structure for a given set of input features^16^. Random forest (RF) is an ensemble of DTs built using different subsets of the dataset to reduce overfitting and noise^17^. Gradient boosting is class of sophisticated ensemble DT algorithms, where individual trees are built and summed sequentially such that the prediction error is minimized during model fitting. Different variants were adopted in this study, including the standard gradient boost (gboost) from the free scikit-learn machine learning library (https://scikit-learn.org/), the eXtreme Gradient Boosting (XGBoost) that iteratively combines multiple weaker base predictors^18^, light gradient boosting machine (lightGBM) by Microsoft Corporation which employs histogram-based DTs grown in a leaf-wise manner^19^, and categorical boosting (CatBoost)^20^ which deals with categorical features. SVM performs classification in higher dimensional feature space where a hyperplane is identified to separate distinct classes^21^. Two linear SVMs were adopted, including support vector classification (SVC), and its variant linearSVC which is more flexible and runs faster. KNN is a classical algorithm which perform classification based on a similarity measure. In hard-voting ensemble learning, denoted here as ensemble learning, the votes for the outcomes from the above algorithms are summed and the predicted class is the one with most votes.

In this study, class sensitive learning (CSL) is applicable to 9 algorithms – XGBoost, lightGBM, CatBoost, SVC, linearSVC, DT, LR, RF and ensemble learning – to counteract class imbalance with the minority class weighted higher^22^, thereby yielding 18 models of the original and the CSL versions. Adding Gboost and KNN, a total of 20 models were built.

### Statistical analysis

Continuous variables were described as median and interquartile ranges, categorical variable as number and percentages as appropriate. We evaluated and compared the performance of each prediction model using Area-under-curve (AUC) of receiver-operating characteristics (ROC) for the training and validation.

### Ethics approval

The study was performed in accordance with relevant guidelines and regulations. All experimental protocols were approved by a The Joint Chinese University-New Territories East Cluster Clinical Research Ethics Committee of Hong Kong, SAR. Informed consent was obtained from all participants involved in the study.

## Results

### Baseline characteristics

Overall, 3129 patients fulfilled the inclusion criteria and were therefore enrolled. The training set, consisting of data from 637 patients undergoing RNU from Asia and the validation set, consisting of 2492 patients from Europe.

Overall, median age was 68 years (61–76), 1959 (62,3%) of patients were male. The proportion of tumor located in the pelvis was similar among the two groups (69.7% (444 patients) in the Asian cohort vs. 64.7% (1613 patients) in European cohort). The detailed baseline characteristics of the training and validation cohorts were listed in Table 1.

**Table 1:** Baseline characteristics of patients enrolled in training (Asian patients) and validation (European patients) cohort.

Feature type	Feature – median (IQR) / *n* (%)	All (*n* = 3129)	Training cohort (*n* = 637)	Validation cohort (*n* = 2492)
patient-related	Gender – Male	1951 (62.3)	270 (42.4)	1681 (67.5)
patient-related	Gender – Female	1178 (37.7)	367 (57.5)	811 (32.5)
patient-related	Age	68 (61–76)	68 (61–76)	68 (61–76)
tumor-related	Grading – G1/Low Grade	127 (4)	112 (17.6)	15 (0.6)
tumor-related	Grading – G2	394 (12.6)	5 (0.8)	389 (15.6)
tumor-related	Grading – G3/High grade	2608 (83.3)	520 (81.6)	2088 (83.8)
tumor-related	pT – pT0	17 (0.5)	2 (0.3)	15 (0.6)
tumor-related	pT – pTa	631 (20.2)	104 (16.4)	527 (21.2)
tumor-related	pT – pTis	55 (1.8)	7 (1.1)	48 (1.9)
tumor-related	pT – pT1	713 (22.8)	158 (24.8)	555 (22.2)
tumor-related	pT – PT2	612 (19.6)	139 (21.8)	473 (19.0)
tumor-related	pT – pT3	945 (30.2)	191 (30.0)	754 (30.2)
tumor-related	pT – pT4	156 (4.9)	36 (5.7)	120 (4.9)
tumor-related	pN – pNx	2179 (69.6)	504 (79.1)	1675 (67.2)
tumor-related	pN – pN0	699 (22.3)	104 (16.3)	595 (23.8)
tumor-related	pN – pN1	236 (7.5)	14 (2.2)	222 (9)
tumor-related	pN – pN2	15 (0.5)	15 (2.4)	
tumor-related	multi-focality – Yes	795 (25.4)	205 (32.2)	590 (23.7)
tumor-related	multi-focality – No	2334 (74.6)	432 (67.8)	1902 (76.3)
tumor-related	CIS – Yes	633 (20.2)	60 (9.4)	573 (23.3)
tumor-related	CIS – No	2496 (79.8)	577 (90.6)	1919 (76.7)
tumor-related	Lymphovascular invasion – Yes	1078 (34.5)	500 (78.5)	578 (23.2)
tumor-related	Lymphovascular invasion – No	2051 (65.5)	137 (21.5)	1914 (76.8)

IQR: Inter Quartile Range, CIS: Carcinoma In Situ.

### Training

The results of each model in terms of AUC for the prediction of each outcome upon training are presented in **Supplementary Table ****1**, the best five models are highlighted for each of the outcome. Overall, LR models seems to achieve the best results, being the number 1 model for prediction of 4/6 outcomes (AUC: 0.85, 0.84, 0.81, 0.77 for CSS-3y, CSS-5y, DFS-3y and OS-5y respectively) and number 2 on the other 2/6 outcomes (OS-3y and DFS-5y).

Regarding OS, the models show results slightly lower than 0,8 in AUC: the best model is SVC for OS-3y [AUC: 0.79 (95% CI 0.7142–0.8630)] and LR for OS-5y [ AUC: 0.77(0 0.7088–0.8398)].

Better results seem to be obtained in predicting the DFS, slightly overcome the threshold of AUC of 0.8, both at 3- and 5-years: the best DFS-3y model is LR [AUC of 0.81 (95% CI 0.7386–0.8816)] while at DFC − 5y prevails LR (CSL) [AUC of 0.80 (95% CI 0.7335–0.8751)].

The outcome showing the overall most promising results in all the trained models is the CSS, with a peak of AUC reaching 0,85: in this case the LR model provides the best results at both CSS-3y [AUC: 0.85 (95% CI 0.7839–0 0.9151)] and CSS-5y [AUC: 0.84 (95% CI 0.7680–0.9070)].

### External validation

The results of each model in terms of AUC for the prediction of each outcome upon validation are presented in **Supplementary Table ****2**, the best five models are highlighted for each of the outcome. Overall, upon validation LR(CSL) models achieved the best results, being the number 1 model for prediction of 3/6 outcomes (AUC: 0.84, 0.79, 0.77 for CSS-3y, OS-3y and OS-5y respectively), followed by LinearSVC(CSL) (AUC: 0.82 and 0.82 for DFS-3y and DFS-5y, respectively).

**Fig. 1:**
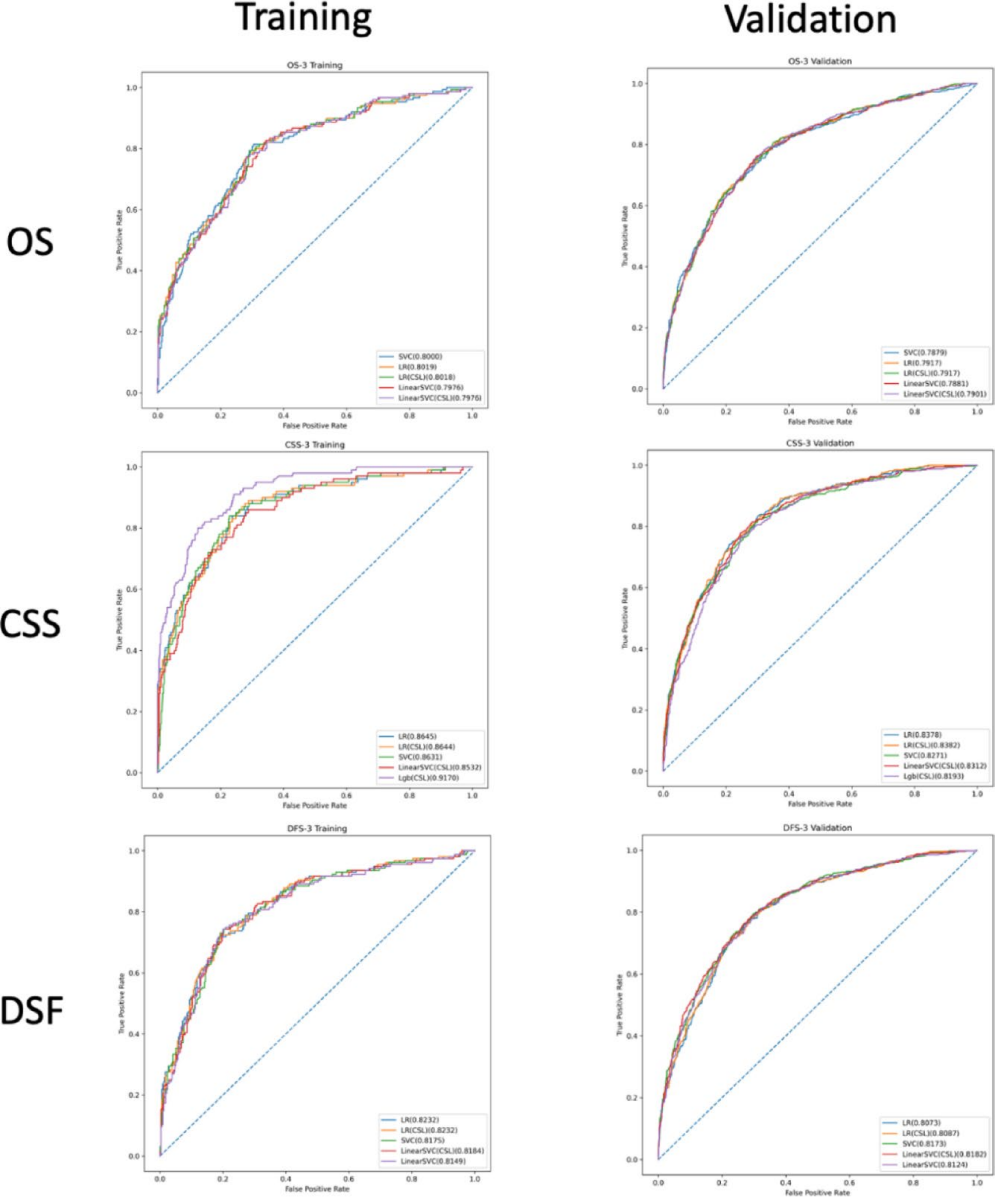
Comparison of the best 5 models upon training and their results upon validation at 3 years. From left to right from top to bottom: OS training and validation at 3 years, CSS training and validation at 3 years, DFS training and validation at 3 years. OS: Overall Survival; CSS: Cancer Specific Survival; DFS: Disease Free Suvival.

**Fig. 2:**
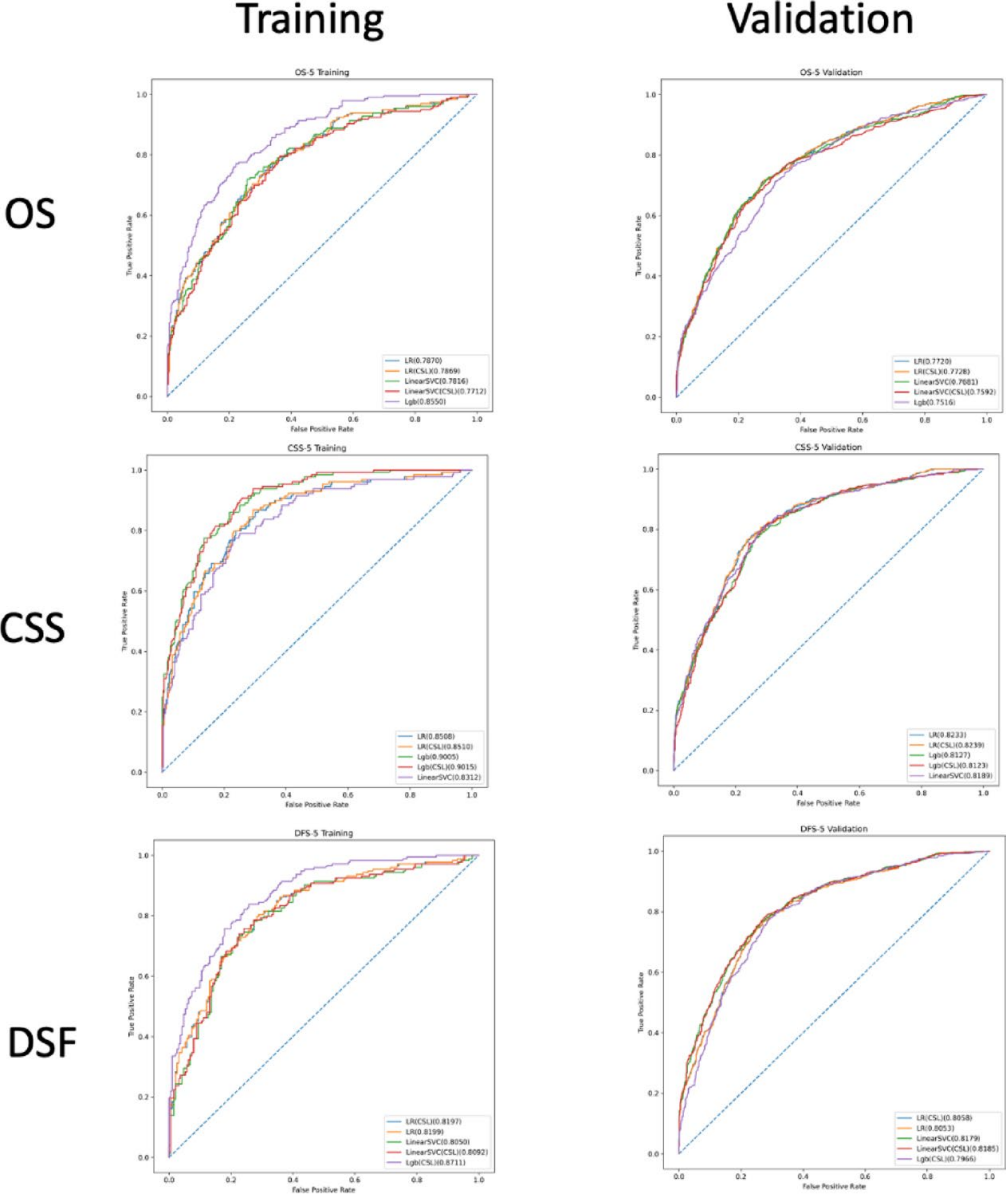
Comparison of the best 5 models upon training and their results upon validation at 5 years. From left to right, from top to bottom: OS training and validation at 5 years, CSS training and validation at 5 years, DFS training and validation at 5 years. OS: Overall Survival; CSS: Cancer Specific Survival; DFS: Disease Free Suvival.

The comparison in AUC of the top 5 models are available in Fig. 1 for prediction of outcomes at 3year and Fig. 2 for 5year, the top 5 model’s AUC details upon training and validation are listed Table 2.

**Table 2:** Top 5 models for prediction of each of the six outcomes, in terms of AUC upon training and Validation.

Outcome	Upon Training	AUC	Upon Validation	AUC
OS – 3 years	SVC	0.7886	LR(CSL)	0.7917
OS – 3 years	LR	0.7883	LR	0.7917
OS – 3 years	LR(CSL)	0.7872	LinearSVC(CSL)	0.7901
OS – 3 years	LinearSVC	0.7815	LinearSVC	0.7881
OS – 3 years	LinearSVC (CSL)	0.7805	SVC	0.7879
OS – 5 years	LR	0.7743	LR(CSL)	0.7728
OS – 5 years	LR(CSL)	0.7739	LR	0.7720
OS – 5 years	LinearSVC	0.7676	LinearSVC	0.7681
OS – 5 years	LinearSVC (CSL)	0.7635	LinearSVC(CSL)	0.7592
OS – 5 years	Lgb	0.7597	Catboost	0.7587
CSS – 3 years	LR	0.8495	LR(CSL)	0.8382
CSS – 3 years	LR(CSL)	0.8471	LR	0.8378
CSS – 3 years	SVC	0.8399	LinearSVC(CSL)	0.8312
CSS – 3 years	LinearSVC(CSL)	0.8377	Catboost	0.8277
CSS – 3 years	Lgb(CSL)	0.8334	SVC	0.8271
CSS – 5 Years	LR	0.8375	SVC	0.8267
CSS – 5 Years	LR(CSL)	0.8362	LR(CSL)	0.8239
CSS – 5 Years	Lgb	0.8260	LR	0.8233
CSS – 5 Years	Lgb(CSL)	0.8241	Catboost	0.8200
CSS – 5 Years	LinearSVC	0.8212	LinearSVC	0.8189
DFS – 3 years	LR	0.8101	LinearSVC(CSL)	0.8182
DFS – 3 years	LR(CSL)	0.8093	SVC	0.8173
DFS – 3 years	SVC	0.8055	LinearSVC	0.8124
DFS – 3 years	LinearSVC(CSL)	0.8002	LR(CSL)	0.8087
DFS – 3 years	LinearSVC	0.7995	LR	0.8073
DFS – 3 years	LR(CSL)	0.8043	LinearSVC(CSL)	0.8185
DFS – 5 years	LR	0.8040	LinearSVC	0.8179
DFS – 5 years	LinearSVC(CSL)	0.7908	LR(CSL)	0.8058
DFS – 5 years	LinearSVC	0.7879	LR	0.8053
DFS – 5 years	Lgb(CSL)	0.7828	Catboost	0.7973

OS: Overall Survival, CSS: Cancer Specific Survival, DFS: Disease Free Survival, AUC: Area Under Curve, CI: Confidence Interval.

Regarding OS, overall the models upon external validation show results slightly higher as compared to training: the best model is LR (CSL) for OS 3y [AUC: 0.79] and OS 5y [ AUC: 0.77].

Better results are obtained in predicting the DFS, slightly overcome the threshold of AUC of 0.8, both at 3- and 5-years: the best DFS-3y model is SVC [AUC of 0.82] while at DFC − 5y prevails LinearSVC(CSL) [AUC of 0.82].

Similar to training, the outcome with the most significant results is the CSS, with a peak of AUC reaching 0,84: the best CSS-3y is LR model [AUC: 0.84], while for CSS-5y best performance is by SVC [AUC: 0.83].

## Discussion

In our study, the use of various ML-supervised models has shown good prediction value of oncological outcomes of UTUC after RNU. Using readily available clinical parameters, ML-supervised models could provide an accurate prediction of prognosis, potentially implementing pTNM staging alone as a guide for postoperative treatment. Among the various experiments, the LR ML-supervised model obtains the best results in predicting CSS at both 3 and 5 years, with a maximum AUC reached of 0.85 and 0.84 respectively upon training, while LR(SVC) is more reliable upon validation, with best results in CSS 3-year. Although we acknowledge that our is not the first attempt to propose a prediction models after RNU, the existing models are not fully comparable: (1) to date, we used one of the largest cohort of patients (*n* = 3129) for UTUC’s prognosis prediction; (2) we intentionally included two set of patients with different ethnicity; (3) we explored the applicability of ML-supervised models in UTUC field; (4) we performed a complete external validation, in fact the only model external validate was the Yates et Al’s model, using 200 bootstrap resamples.

Adjuvant therapies are invasive and burdened by toxicity, especially in the setting of single-kidney patients who might not even need it if better prediction tools exist. Numerous efforts have been made to generate predictive models of UTUC postoperative prognosis. Despite this, there is a lack of validation which makes these models still not reliable in clinical practice and none is yet recommended with strong evidence by current European guidelines. The POUT trial^3^, which is currently interested in the validation of adjuvant chemotherapy after RNU for UTUC, uses only pTNM staging data for selection porpoise, including pT2–T4 pN0–N3 M0 or pT any N1–3 M0; however his preliminary subgroup analysis demonstrated large variability in the benefits of patients undergoing adjuvant chemotherapy, underlining the need for a better stratification strategy after RNU, taking into account additional features.

Several prognosis prediction nomograms have been proposed, these tools exceed AJCC/TNM staging for prognosis of survival in internal validation. Among these, two studies^4,6^ include UTUC patients undergoing surgery regardless of RNU or other conservative surgery; Ku et Al^11^ limited to an external validation study, while instead Krabbe et al.^7^used different outcomes from ours study as per Relapse free survival, are therefore not comparable. Overall, four models^5,8–10^ are comparable, however, one of them use the old WHO 1973 grading system^8^. Those nomograms variously used 7 different independent prognostic factors (Age, pT, LVI, Location, CIS, Architecture, pN), with Cha’s model being the more comprehensive (7 features) followed by Seisen (6 features) and Roupret (5 features). All of the models assessed 5y-CSS; Cha’s model additionally assessed 2y-CSS; none exceeds the trade-off of 0.81 in terms of AUC for the prediction of CSS, neither in the training nor in the internal validation set. This support the hypothesis that the ML could implement existing models.

Furthermore, our study represents the first attempt to generate a model that can be reliable in more than one single ethnicity: most of this nomograms didn’t take into account the existing differences in Asian patients, who seem to present more advanced and higher-grade diseases compared to other ethnicities^12^. This could be explained with differences in genetic and epigenetic factors such as environmental and occupational exposures, lifestyle choices as well as socioeconomic factors^23^. Aiming to move towards a race-conscious medicine, keeping in mind that as suggested by Cardena et al.^24^ clinical research should be used to examine structural barrier, we decided use two set of patients with different ethnicity, rather than using race as a proxy for biology. Our models are therefor tested to both European and Asian patients and can be reliable regardless the origin of the patient.

Various machine learning techniques have been used in the field of Urology, most of them within the lower urinary tract setting: (1) regarding radiomics, AI have been implemented, capable of distinguishing between bladder tumor and normal bladder at multi parametric magnetic resonance imaging (mpMRI)^25^ or determining the stage of bladder cancer at Computed Tomography (CT)^26^; (2) in terms of prognosis, the only experience derives from Lam et al. and Wang et al., who used clinicopathological evidence to create and test a significant number of AI algorithms to estimate the 5-year survival after radical cystectomy^27,28^. To date, this is the first experience investigating the possible application of ML-supervised algorithms to the UTUC and in particular to predict prognosis after RNU.

Lastly, or model may help clinicians in stratifying patients with UTUC, addressing the challenge of understanding clinical aggressiveness based on baseline characteristics of this specific tumors. Not only it can be used to increment follow-up strategies in patients with high risk of recurrence, but also can be used to stratify potential candidate to adjuvant and subsequential therapies.

This study has several limitations. First, its nature as a multicenter study may have introduced inconsistencies in surgical skills, type of bladder cuff performed, use or not of intra- or perioperative mitomycin, neoadjuvant use of chemotherapy and pathological diagnoses. Second, since the cohort straddles 2004, the use of two different pathological gradings may have influenced the algorithms. Furthermore, 2 patients on training and 15 patients on validation cohort had a pT0 diagnosis at final histopathological specimen: even if this may reflect real world data, on the other hand the prognosis for those patients is by definition excellent. Moreover, there is a non-negligible difference in gender representation among the two cohorts: thus, due to the different underneath biology, may influence response to treatments and prognosis. Lastly, the lack of centralized pathological revisions of imaging and specimen could introduce a bias.

## Conclusions

ML is a promising technology in the field of UTUC. Our model achieve favorable results in terms of prediction of prognosis after RNU, especially in terms of CSS at 3 and 5 years, moreover is the first model of prognosis taking into account the differences in epidemiology existing between European and Asian patients. Further clinical validation and verification of its reliability for the case selection of adjuvant therapy are needed to assess its use in clinical practice linked to clinical decision making.

## Supplementary Information

Below is the link to the electronic supplementary material.

Supplementary Material 1

Supplementary Material 2

Supplementary Material 3

## Data Availability

Data are available for bona fide researchers who request it from the authors. Please contact the corresponding author for related requests.
