# Supplementary material for: Training and external validation of machine learning supervised prognostic models of upper tract urothelial cancer (UTUC) after nephroureterectomy
Source: Sci Rep. 2026 Jan 22;16:2847. doi: 10.1038/s41598-025-29043-w (PMC12827265; doi:10.1038/s41598-025-29043-w)
Supplement: Supplementary file 3 — Supplementary Material 3 [file 41598_2025_29043_MOESM3_ESM.docx]

**Supplementary file**

***Legends for supplementary file***

**Supplementary table 1** shows the best five models are highlighted for each of the outcome. LR models is associated with the best results, being the number 1 model for prediction of 4/6 outcomes (AUC: 0.85, 0.84, 0.81, 0.77 for CSS-3y, CSS-5y, DFS-3y and OS-5y respectively) and number 2 on the other 2/6 outcomes (OS-3y and DFS-5y).

**Supplementary table 2** shows the results of each model in terms of AUC for the prediction of each outcome upon validation. LR(CSL) models achieved the best results, being the number 1 model for prediction of 3/6 outcomes (AUC: 0.84, 0.79, 0.77 for CSS-3y, OS-3y and OS-5y respectively), followed by LinearSVC(CSL) (AUC: 0.82 and 0.82 for DFS-3y and DFS-5y, respectively).
